# Elevated troponin and myocardial infarction in the intensive care unit: a prospective study

**DOI:** 10.1186/cc3816

**Published:** 2005-09-28

**Authors:** Wendy Lim, Ismael Qushmaq, Deborah J Cook, Mark A Crowther, Diane Heels-Ansdell, PJ Devereaux

**Affiliations:** 1 Research Fellow, Department of Medicine, McMaster University, Hamilton, Ontario, Canada; 2 Professor, Departments of Medicine and Clinical Epidemiology & Biostatistics, McMaster University, Hamilton, Ontario, Canada; 3 Associate Professor, Department of Medicine, McMaster University, Hamilton, Ontario, Canada; 4 Statistical Analyst, Department Clinical Epidemiology & Biostatistics, McMaster University, Hamilton, Ontario, Canada; 5 Assistant Professor, Departments of Medicine and Clinical Epidemiology & Biostatistics, McMaster University, Hamilton, Ontario, Canada

## Abstract

**Introduction:**

Elevated troponin levels indicate myocardial injury but may occur in critically ill patients without evidence of myocardial ischemia. An elevated troponin alone cannot establish a diagnosis of myocardial infarction (MI), yet the optimal methods for diagnosing MI in the intensive care unit (ICU) are not established. The study objective was to estimate the frequency of MI using troponin T measurements, 12-lead electrocardiograms (ECGs) and echocardiography, and to examine the association of elevated troponin and MI with ICU and hospital mortality and length of stay.

**Method:**

In this 2-month single centre prospective cohort study, all consecutive patients admitted to our medical-surgical ICU were classified in duplicate by two investigators as having MI or no MI based on troponin, ECGs and echocardiograms obtained during the ICU stay. The diagnosis of MI was based on an adaptation of the joint European Society of Cardiology/American College of Cardiology definition: a typical rise or fall of an elevated troponin measurement, in addition to ischemic symptoms, ischemic ECG changes, a coronary artery intervention, or a new cardiac wall motion abnormality.

**Results:**

We screened 117 ICU admissions and enrolled 115 predominantly medical patients. Of these, 93 (80.9%) had at least one ECG and one troponin; 44 of these 93 (47.3%) had at least one elevated troponin and 24 (25.8%) had an MI. Patients with MI had significantly higher mortality in the ICU (37.5% versus 17.6%; *P *= 0.050) and hospital (50.0% versus 22.0%; *P *= 0.010) than those without MI. After adjusting for Acute Physiology and Chronic Health Evaluation II score and need for inotropes or vasopressors, MI was an independent predictor of hospital mortality (odds ratio 3.22, 95% confidence interval 1.04–9.96). The presence of an elevated troponin (among those patients in whom troponin was measured) was not independently predictive of ICU or hospital mortality.

**Conclusion:**

In this study, 47% of critically ill patients had an elevated troponin but only 26% of these met criteria for MI. An elevated troponin without ischemic ECG changes was not associated with adverse outcomes; however, MI in the ICU setting was an independent predictor of hospital mortality.

## Introduction

Cardiac troponin is specific to the myocardium, and levels in the serum rise 3–4 hours after the occurrence of cardiac symptoms in patients with acute myocardial infarction (MI) [1]. Because of its high sensitivity and specificity, elevated levels of troponin indicate myocardial damage but not the mechanism of damage. The diagnosis of MI has thus evolved following the introduction of routine troponin testing, resulting in the redefinition of MI by the joint European Society of Cardiology (ESC)/American College of Cardiology (ACC) in 2000 [2]. Based on this consensus document, any amount of myocardial damage, as detected by serum troponin and associated with evidence of ischemia, should be considered an MI.

It is increasingly recognized that elevated troponin levels occur in many patients who do not have evidence of flow-limiting coronary artery disease [3,4]. In addition to nonthrombotic cardiac conditions (myocardial contusion, infiltrative myocardial diseases), nonthrombotic noncardiac diagnoses (sepsis, pulmonary embolism, stroke, renal failure) are also associated with elevated levels of troponin. Given this observation, it is generally considered inappropriate to use elevated troponin levels as the only diagnostic criterion for MI [5]. The ESC/ACC consensus document [2] defines MI not only based on a typical rise and fall in biomarkers but also requires the presence of one of the following: ischemic symptoms, electrocardiogram (ECG) changes consistent with myocardial ischemia, or need for a coronary artery intervention.

Use of a 12-lead ECG recording is the most common and widely available method for assessing for the presence of myocardial ischemia in the intensive care unit (ICU). Continuous ECG recordings and use of cardiac echocardiography for wall motion abnormalities, while available, have not been well studied in these patients [6]. Although coronary angiography is considered the reference standard diagnostic test for coronary heart disease, it is not feasible to perform angiography in all critically ill patients with elevated troponin levels. Therefore, because of limitations in other diagnostic tests, the use of intermittent 12-lead ECG in combination with monitoring troponin levels is the practical approach that most physicians use to diagnose MI in the ICU.

In the ICU, the diagnosis of MI is challenging for many reasons. Symptoms of MI in critically ill patients may be masked by sedative or analgesic medications; these patients are also frequently unable to communicate ischemic symptoms because of endotracheal intubation or coma. Furthermore, because elevated troponin levels occur in critically ill patients without evidence of myocardial ischemia, the interpretation of an elevated troponin value is variable among clinicians and is often uncertain. More importantly, elevated troponin levels predict a poor prognosis in patients with acute coronary syndromes [7-12] and may also predict adverse outcomes in patients admitted to the ICU. In a medical ICU, patients with elevated troponin T or I admitted with nonacute coronary syndrome diagnoses exhibited a fourfold higher mortality (22.4 versus 5.2%; *P *< 0.018) [3]. In surgical ICU patients, moderate elevations in troponin I (0.4–2.0 μg/l) were associated with higher mortality (rates ranging from 12.4% to 38.4%, depending on the degree of troponin elevation) than in patients with normal troponin levels (3.3%); longer hospital and ICU lengths of stay were also found in patients with elevated troponin [13].

Based on these studies, the association between elevated troponin and adverse outcomes remains uncertain because univariable analyses were conducted, which does not account for the likelihood that patients with elevated troponin levels likely have other reasons for a worse outcome. Studies that used multivariable analyses are limited but have suggested that an elevated troponin I level is associated with increased cardiac events in a medical-surgical ICU [6], and is associated with increased in-hospital death in ICU patients with exacerbations of chronic obstructive pulmonary disease [14] and left ventricular dysfunction in patients with septic shock [15].

The interpretation of elevated troponin levels during critical illness remains unclear. In the ICU some patients with elevated troponin values and nonspecific ECG changes are considered to have suffered an MI and are treated with anti-ischemic, antiplatelet, and anticoagulant therapies, whereas others are considered to have an alternative explanation for the troponin rise and do not receive any of these therapies. As a first step toward exploring these issues, we performed a prospective cohort study in medical-surgical ICU patients to examine the frequency of MI, in which the troponin levels were examined in relation to 12-lead ECGs to diagnose MI. We also examined whether elevated troponin levels and MI were associated with the outcomes of hospital and ICU mortality and length of stay.

## Materials and methods

### Patients

In this prospective cohort study, we included all consecutive patients admitted to the ICU at St Joseph's Hospital (Hamilton, Ontario, Canada) from 12 July to 12 September 2004. All aspects of patient management were at the discretion of the ICU team, which was unaware of the study in order to eliminate any influence on troponin or ECG test ordering. This study was approved by our institutional research ethics board, which waived the need for informed consent for this noninterventional audit with no influence on clinical decision making.

### Setting

The ICU at St Joseph's Hospital is a 15-bed, university affiliated medical-surgical ICU. Although the hospital has a cardiac care unit for patients with primary cardiac diagnoses or requiring telemetry, such patients also requiring mechanical ventilation and those receiving inotropes or vasopressors are admitted to the ICU. The ICU is staffed by critical care physicians and physicians in training.

### Data collection

We collected all 12-lead ECGs, troponin measurements and echocardiograms performed during the ICU admission. The frequency and timing of all troponin measurements, ECGs and echocardiography was determined by the ICU team based on their clinical judgment, and appropriate follow up of abnormal results was also left to the discretion of the ICU team. Although not mandated, many patients in the ICU have screening troponin levels and ECG recordings routinely performed within 24 hours of ICU admission. We also collected information on patient demographics, baseline risk factors for cardiac disease, need for advanced life support (mechanical ventilation, inotropes or vasopressors, and dialysis), cardiac medications, ICU and hospital mortality, and length of ICU and hospital stay.

#### Electrocardiography

Twelve-lead ECGs (PageWriter, Hewlett-Packard, Palo Alto, CA, USA) were obtained at the direction of the ICU team as clinically indicated. ECGs were performed by a technologist during the day, and by the ICU bedside nurse in emergencies and during the evenings and weekends. We removed all patient identifiers from the ECG before their interpretation by the investigators. To replicate clinical practice, the computer generated ECG interpretation printed on the ECGs was not removed.

#### Troponin measurements

Blood samples for troponin T measurements were drawn into EDTA tubes, and plasma for sample analysis was obtained following centrifugation of whole blood at 1,500 *g *for 15 min. Troponin T was measured using an electrochemiluminescence immunoassay (Roche Modular analytics E170 [Elecsys module] immunoassay analyzer; Roche Diagnostics, Indianapolis, IN, USA). The analytical sensitivity (lower detection limit) of this assay is 0.01 μg/l.

### Interpretation of results

#### Analysis of troponin levels

In accordance with the US National Academy of Clinical Biochemistry draft guidelines on biomarkers of the acute coronary syndrome and heart failure, a single cutpoint at the lowest analytical value with 10% coefficient of variation was used [16]. Using these guidelines, cardiac troponin T values above 0.04 μg/l were considered evidence of myocardial necrosis and levels of 0.04 μg/l or less were considered to represent no evidence of myocardial necrosis.

#### Classification of myocardial infarction

The definition of MI in the ICU was adapted from the joint ESC/ACC redefinition of acute MI [2]. The consensus document defines MI through pathologic findings, or the presence of a typical rise and gradual fall in troponin or a more rapid rise and fall in creatine kinase-MB with one of the following: ischemic symptoms, development of pathologic Q waves on ECG, ischemic ECG changes (ST-segment elevation or depression), or a coronary artery intervention.

We made two adaptations to the proposed criteria. First, we adapted the biomarker criterion because the troponin rise can be missed in the absence of patient communication, and an elevated troponin can be discovered following the peak level and after an event has occurred. In addition, troponin can remain elevated for up to 14 days [1] and in practice is not always remeasured to ensure that it is decreasing. Consequently, we accepted either a typical rise or a typical fall in troponin to satisfy this criterion. Second, although the summary section of the consensus document did not include imaging techniques as a criterion for diagnosing MI, it is included in the text. We introduced the presence of new or presumed new cardiac wall motion abnormalities on transthoracic echocardiography or radionuclide imaging, in combination with elevated biomarkers, to diagnose MI in the ICU. Without this additional echocardiogram criterion, physicians might miss the diagnosis of MI in patients who have suffered an MI. This may occur because ICU patients with an elevated troponin are invariably unable to communicate ischemic symptoms, and some may have an uninterpretable ECG, a chronic left bundle branch block, or infarction in a territory of the ECG that has low sensitivity for MI.

Using these *a priori *criteria, two investigators (IQ, DJC) were provided with all available 12-lead ECGs and troponin measurements for each patient, and echocardiogram results. With the investigators blinded to each other's assessments, patients were then independently classified as having MI or no MI during their ICU stay [2].

### Statistical analysis

We report continuous data as mean and standard deviation or median and interquartile range (IQR), as appropriate. We report proportions with 95% confidence intervals (CIs) for binary data. We compared continuous variables using unpaired t-tests or the Wilcoxon two-sample rank sum test, and dichotomous variables using Fisher's exact test. In multivariable regression analyses, we adjusted for Acute Physiology and Chronic Health Evaluation (APACHE) II score and advanced life support (mechanical ventilation, inotropes or vasopressors, and hemodialysis at any time in the ICU) in order to examine the association between MI and both ICU and hospital mortality. Because troponin is a key component of the diagnosis of MI but may be increased in conditions other than MI, we examined the independent additional risk for death associated with elevated troponin level by including it in a sensitivity analysis in the final model of hospital mortality. Associations are expressed using odds ratios (ORs) and 95% CIs.

## Results

### Patients

**Figure 1:**
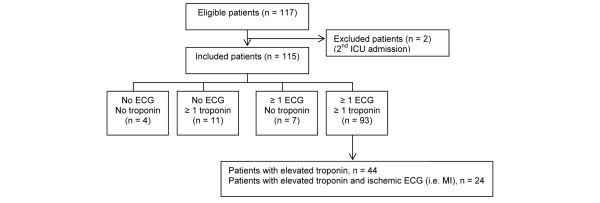
Electrocardiogram and troponin measurement frequency for enrolled patients. ECG, electrocardiogram; MI, myocardial infarction.

We screened 117 admissions to the ICU during the study period. Two patients were admitted twice, and only their first ICU admission was considered, resulting in enrolment of 115 patients in the study. The frequency of ECG recordings and troponin measurements is shown in Fig. 1. Of the 115 patients, 93 (80.9%) patients had at least one ECG performed and one troponin measurement during ICU admission, seven (6.1%) had at least one ECG performed but no troponin measurement, 11 (9.6%) had at least one troponin measurement but no ECG performed, and four (3.5%) had neither an ECG performed nor troponin measurement. Patients had a median of 2 (IQR 1–4) ECGs during their ICU stay. For 23 patients, 28 echocardiograms were performed.

Patient characteristics are summarized in Table 1. The mean ± standard deviation age of the patients was 64.1 ± 17.2 years and they had a APACHE II score of 21.9 ± 9.8; 61 (53.0%) were female. Most admissions were medical (72.2%), and most patients (62.6%) were mechanically ventilated (invasively or noninvasively) at the time of enrolment.

Excluding the 22 patients without both a troponin level and an ECG performed, the frequency of MI based on available ECGs and troponin measurements was 25.8%, occurring in 24 patients. Twenty-one patients sustained a non-ST-segment elevation MI whereas three sustained an ST-segment elevation MI. Patients diagnosed with MI were more likely to have underlying insulin-requiring diabetes mellitus and peripheral vascular disease than were those patients without MI. No patients with MI had ischemic chest pain symptoms, and no patients with MI had this diagnosis made based on angiography, percutaneous coronary intervention, or autopsy. The median (IQR) troponin level in patients with MI was significantly higher (0.26 μg/l [0.16–0.72 μg/l]) than in patients without MI (<0.04 μg/l [<0.04–0.05 μg/l]; *P *< 0.0001).

Table 2 shows the frequency of morbidity and mortality outcomes. Both ICU and hospital mortality were significantly higher in patients diagnosed with MI than in those without MI (37.5% versus 17.6%, *P *= 0.05; and 50.0% versus 22.0%, *P *= 0.01, respectively). We found no difference between patients with and without MI with respect to the duration of mechanical ventilation or duration of ICU or hospital stay. These outcomes were no different between patients with ST-segment elevation MI (*n *= 3) and non-ST-segment elevation MI (*n *= 21; data not shown).

Table 3 summarizes factors associated with ICU and hospital mortality in the univariable and multivariable regression analyses. Factors independently associated with ICU mortality were APACHE II score (OR 2.70, 95% CI 1.27–5.72) and need for inotropes or vasopressors (OR 6.12, 95% CI 1.31–28.68). Factors independently associated with hospital mortality were APACHE II score (OR 2.37, 95% CI 1.21–4.63), need for inotropes or vasopressors (OR 4.76, 95% CI 1.27–17.82) and a diagnosis of MI (OR 3.22, 95% CI 1.04–9.96). When troponin values were added to the latter model, it was not significant in the multivariable analysis but was significant in the univariable analysis; for troponin values of 0.04–1.0 μg/l and ≥1.1 μg/l, the ORs were 2.99 (95% CI 1.23–7.23) and 9.33 (95% CI 1.53–56.93), respectively, compared with the normal reference range (<0.04 μg/l).

## Discussion

Because cardiac troponin is a sensitive and specific measure of myocardial necrosis, it is the preferred biomarker for use in the diagnosis of acute MI. Although an elevated troponin indicates myocardial necrosis, it does not always indicate MI. Thus, in the ICU setting, where elevated troponin is frequently observed, additional evidence of myocardial ischemia can be obtained by using a 12-lead ECG. In this single centre prospective cohort study of predominantly medical ICU patients, 47% of critically ill patients had at least one elevated troponin measurement but only 26% met diagnostic criteria for MI based on a typical rise or fall in elevated troponin measurements and ischemic changes on a 12-lead ECG, with ECGs performed as clinically indicated. Patients with MI had significantly higher troponin levels than did those without MI. Patients who were diagnosed with MI had twofold increased rates of ICU and hospital mortality. The presence of an elevated troponin measurement alone was not associated with adverse outcomes, but the presence of MI was independently predictive of hospital mortality.

The incidence and prevalence rates of elevated levels of troponin cited in the literature vary widely, ranging from 15% to 70% of patients [17-19]. Recent studies have examined the frequency of elevated troponin levels excluding those patients with underlying coronary heart disease [3]; a 55% prevalence of elevated levels of troponin was reported, of which 72% of patients with an elevated troponin did not have flow-limiting coronary artery disease based on stress echocardiography or autopsy. The variability in frequency rates observed in our study and other studies is likely due to the heterogeneous nature of ICU populations and the threshold at which a troponin measurement is considered positive. The current troponin threshold recommended by the ESC/ACC has been defined for noncritically ill populations, and whether this threshold differs in the ICU setting is unknown. Furthermore, the various troponin assays are not standardized and, although it is recommended that levels exceeding the 99th percentile be considered positive, this level varies according to manufacturer [20].

It is important to recognize that although a considerable number of ICU patients have elevated troponin measurements, and elevated troponin measurements are specific for myocardial necrosis, troponin itself does not distinguish between ischemic and nonischemic etiologies of myocardial injury. Interpretation of elevated troponin levels in the ICU must be considered in the context of the patient's symptoms (frequently limited in the ICU) or correlated with ECG findings or other imaging modalities. Most studies have examined elevated troponin levels in the critically ill in isolation, and it is unclear what proportion of patients have actually suffered an MI. One study evaluated troponin with ECGs in 34 consecutive critically ill patients who were mechanically ventilated and underwent thoracic or vascular surgery [21]. It found that 11 patients (32%) had elevated troponin levels, and ECGs were available in 10 patients. Four patients (12%) had ST-segment elevation or depression, meeting criteria for MI; three patients had nonspecific changes and three had no ECG changes. Another study used continuous 12-lead telemetry monitoring in 76 patients admitted with noncardiac conditions [6]. An elevated troponin level was found in 12 patients (15.8%), and six of these patients had transient ischemic events (mainly ST-segment depression) on telemetry.

The importance of a diagnosis that does not alter a patient's prognosis is questionable. Therefore, potentially the most important reason to identify critically ill patients with elevated troponin as having an MI or not having an MI is that the prognosis of these patients may be different. In the noncritically ill population, elevated troponin levels are an independent prognostic marker for short-term and long-term outcomes in patients with acute coronary syndromes. In the ICU several studies have reported that elevated troponin levels are associated with adverse outcomes. Elevated troponin I is a predictor of mortality in medical-surgical ICU patients [17-19], including those without acute coronary syndromes [3], and in ICU patients with early sepsis [22], acute exacerbations of chronic obstructive pulmonary disease [14], pulmonary embolism [23] and following cardiac surgery [24-26]. In surgical ICU patients, troponin is a predictor of mortality and longer length of ICU and hospital stay [13]. However, most studies have examined troponin alone and did not examine prognosis in relation to those patients who had associated ECG changes (i.e. patients with MI).

One retrospective study examined the degree of troponin elevation in relation to prognosis [13], and among the patients with recognized MI mortality was 13.6% in those with moderate elevations of troponin I (2.0–10.0 μg/l) and 32.4% in patients with troponin I above 10.0 μg/l. Like our study, this was limited in that there was no screening; it had a retrospective design, and it was unclear how the diagnosis of MI was made. Although we found that MI was predictive of hospital mortality, it was not predictive of other morbidity outcomes, including the duration of mechanical ventilation and ICU and hospital stays. This may be attributable to the relatively small number of patients included in our study or it may be an artefact of the distribution of some early deaths in this cohort. Similarly, predictors of ICU and hospital mortality, including need for life-saving therapies (hemodialysis, mechanical ventilation), were not significant in the multivariable analysis, which may relate to the distribution of risk factors (hemodialysis being infrequent and mechanical ventilation being common) in a study of this size.

Identification of those critically ill patients with MI has several treatment implications. In noncritically ill patients, patients with elevated troponin levels and acute MI benefit from antithrombotic therapy [27-30]. Critically ill patients who also have elevated troponin and acute MI would also be expected to benefit from these therapies, but those patients who have elevated troponin without MI may not benefit and in fact may be harmed. Furthermore, critically ill patients with ST-segment elevation MI should be distinguished from those with non-ST-segment elevation MI because the former warrants urgent revascularization (or thrombolysis, although this is not always an option for ICU patients). We did not detect differences in outcome in patients diagnosed with ST-segment elevation or non-ST-segment elevation MI, although our analysis was underpowered to detect such differences.

Not only has recognition of MI been poorly studied but also the impact of antithrombotic and anti-ischemic agents has not been well documented in these patients. In one study, surgical ICU patients with moderate elevations in troponin I (2.0–10.0 μg/l, and not necessarily diagnosed with MI) who were treated with β-blockers and aspirin were reported to have lower mortality than patients with the same range of troponin elevation who did not receive these therapies [13]. However, findings from this retrospective study should be cautiously interpreted because selection bias might have resulted in patients who were less critically ill receiving β-blockers and aspirin (e.g. patients without a coagulopathy and not requiring β-agonist infusions).

The strengths of our study include use of *a priori *definitions for MI, and duplicate assessment by two independent investigators to classify events. Determination not only of patients with elevated troponin levels but also of those with MI provides relevant information not previously reported. However, there are several important limitations to the study. First, although ECG has been reported to be more sensitive for detecting myocardial ischemia than conventional ICU monitoring [30], the ECG itself has limitations. The conventional ECG is not very sensitive for detecting infarction in certain locations (posterior) [32], and not all patients who have myocardial necrosis exhibit ECG changes [2]. In addition, uninterpretable ECGs occur among patients who are pacemaker dependent or have left bundle branch blocks, in whom acute changes cannot be detected using a standard 12-lead ECG. A second limitation is that systematic screening of all patients with troponin and ECG recordings was not performed in this study, and hence we cannot determine the true prevalence and incidence of elevated troponin and MI. Finally, there is currently no consensus on the appropriate diagnosis of MI in critically ill patients, whose ability to communicate may be severely limited and in whom diagnostic tests have not been vigorously evaluated. Our results may have differed if we required two or more elevated troponin measurements to meet the biomarker criterion, or if we required two or more ECGs demonstrating evolving changes [5].

The utility of screening for MI in the ICU population has not been studied. In view of the prognostic and possible therapeutic implications of establishing a diagnosis of MI in the critically ill, use of noninvasive tests – including troponin and ECG – may be a reasonable approach. In contrast to the noncritically ill population, limitations in the ability of patients to communicate ischemic symptoms and the use of vasopressors and mechanical ventilation are unique to the ICU and may require the use of alternate methods of diagnosis. However, we cannot recommend for or against systematic troponin screening based on our study. The appropriate use of these tests and other diagnostic methods, including echocardiography in a screening mode, must be properly evaluated in well designed prospective studies.

## Conclusion

In summary, elevated troponin levels are common in critically ill patients, but not all patients with elevated levels have MI. Almost half of the patients admitted to this general medical-surgical ICU (consisting mainly of medical patients) had elevated troponins during their ICU stay, and approximately 26% of patients were found to have an MI. Patients diagnosed with MI in the ICU based on elevated troponin levels and ischemic ECG changes had higher ICU and hospital mortality, and MI was independently predictive of hospital mortality. However, we did not find that an elevated troponin alone (among those patients who had troponin measurements during their ICU admission) was associated with adverse outcomes. Screening with troponin levels in the ICU should not be done in isolation because ECG is necessary to interpret abnormal levels. Future studies should determine the prognostic importance of elevated troponin in the ICU by more comprehensively examining other diagnoses and their consequences, and by evaluating the role of other modalities (i.e. echocardiography, radionuclide imaging, magnetic resonance imaging) to diagnose MI. Second, the role of antithrombotic and anti-ischemic agents in critically ill patients with troponin elevation requires evaluation in large prospective randomized controlled trials to determine the appropriate management of these patients.

## Key messages

• 	The diagnosis of MI in the ICU is usually dependent on elevated troponin levels and ischemic changes on a 12-lead ECG or a new wall motion abnormality on echocardiography, because ICU patients are usually unable to communicate chest pain symptoms as a result of administration of narcotics or sedatives, or decreased level of consciousness.

• 	In this single centre cohort study, 47% of predominantly medical critically ill patients had at least one elevated troponin measurement, but only 26% of these patients had MI.

• 	An elevated troponin level alone is not an independent predictor of ICU or hospital mortality.

• 	Patients with MI had a significant twofold increase in risk for ICU and hospital mortality compared with patients without MI.

• 	After adjusting for APACHE II score and inotrope or vasopressor use, development of MI in the ICU setting was an independent predictor of hospital mortality.

## Abbreviations

ACC = American College of Cardiology; APACHE = Acute Physiology and Chronic Health Evaluation; CI = confidence interval; ECG = electrocardiogram; ESC = European Society of Cardiology; ICU = intensive care unit; IQR = interquartile range; MI = myocardial infarction; OR = odds ratio.

## Competing interests

The author(s) declare that they have no competing interests.

## Authors' contributions

WL, DC, MC, PD, and IQ obtaining funding for the study. DC, IQ, and WL conceived and designed the study. IQ, WL, and DC collected data. DH-A, WL, and DC conducted statistical analysis. WL, DC and DH-A drafted the report, and PD, MC and IQ critically revised the manuscript. DC was guarantor.

## Figures and Tables

**Table 1:** Patient clinical characteristics

	Total (*n *= 115)	MI (*n *= 24)	No MI (*n *= 91)	*P *value
Age (years; mean ± SD)	64.1 ± 17.2	62.8 ± 18.3	64.4 ± 17.0	0.69
Sex (*n*; % female)	61 (53.0)	13 (54.2)	48 (52.7)	1.00
APACHE II score (mean ± SD)	21.9 ± 9.8	26.3 ± 10.4	20.7 ± 9.3	0.01
Medical (*n *[%])	83 (72.2)	20 (83.3)	63 (69.2)	0.21
Epidural (*n *[%])	12 (10.4)	0 (0.0)	12 (13.2)	0.07
Past medical history (*n *[%])				
Smoker	35 (30.4)	5 (20.8)	30 (33.0)	0.32
Hypertension	59 (51.3)	14 (58.3)	45 (49.5)	0.50
Diabetes (oral agent)	9 (7.8)	3 (12.5)	6 (6.6)	0.39
Diabetes (insulin)	17 (14.8)	10 (41.7)	7 (7.7)	<0.001
Hypercholesterolemia	14 (12.2)	2 (8.3)	12 (13.2)	0.73
Angina	15 (13.0)	3 (12.5)	12 (13.2)	1.00
Myocardial infarction	25 (21.7)	9 (37.5)	16 (17.6)	0.05
Congestive heart failure	14 (12.2)	5 (20.8)	9 (9.9)	0.17
Peripheral vascular disease	10 (8.7)	7 (29.2)	3 (3.3)	<0.001
Transient ischemic attack	4 (3.5)	2 (8.3)	2 (2.2)	0.19
Stroke	8 (7.0)	3 (12.5)	5 (5.5)	0.36
Baseline life support interventions (*n *[%])				
Ventilation				
Invasive mechanical ventilation	67 (58.3)	16 (66.7)	51 (56.0)	0.49
Noninvasive mechanical ventilation	5 (4.3)	2 (8.3)	3 (3.3)	0.28
Inotropes and vasopressors				
Epinephrine	2 (1.7)	1 (4.2)	1 (1.1)	0.38
Dopamine	10 (8.7)	3 (12.5)	7 (7.7)	0.43
Norepinephrine	19 (16.5)	5 (20.8)	14 (15.4)	0.54
Dobutamine	4 (3.5)	2 (8.3)	2 (2.2)	0.19
Phenylephrine	18 (15.7)	9 (37.5)	9 (9.9)	0.003
Vasopressin	5 (4.3)	3 (12.5)	2 (2.2)	0.06
Hemodialysis				
Intermittent dialysis	14 (12.2)	4 (16.7)	10 (11.0)	0.49
Continuous renal replacement therapy	0 (0.0)	0 (0.0)	0 (0.0)	-

APACHE, Acute Physiology and Chronic Health Evaluation; MI, myocardial infarction; SD, standard deviation.

**Table 2:** Frequency of morbidity and mortality outcomes

	MI (*n *= 24)	No MI (*n *= 91)	*P *value
Duration of mechanical ventilation (days; median [IQR])	2 (1–6)	2 (0–5)	0.32
Duration of ICU stay (days; median [IQR])	4.5 (2–8)	4 (2–6)	0.62
ICU mortality (*n *[%])	9 (37.5)	16 (17.6)	0.05
Duration of hospital stay (days; median [IQR])	18 (4.5–41)	12 (7–21)	0.34
Hospital mortality (*n *[%])	12 (50.0)	20 (22.0)	0.01

ICU, intensive care unit; IQR, interquartile range; MI, myocardial infarction.

**Table 3:** Predictors of intensive care unit and hospital mortality

Predictor	ICU mortality (OR [95% CI])		Hospital mortality (OR [95% CI])	
	
	Univariable	Multivariable	Univariable	Multivariable
APACHE II score (10-point increment)	3.92 (2.08–7.40)	2.70 (1.27–5.72)	3.10 (1.82–5.30)	2.37 (1.21–4.63)
Mechanical ventilation	6.14 (1.71–21.97)	0.69 (0.11–4.18)	3.66 (1.36–9.82)	0.56 (0.13–2.46)
Inotropes or vasopressors	8.23 (2.95–23.00)	6.12 (1.31–28.68)	6.10 (2.50–14.89)	4.76 (1.27–17.82)
Hemodialysis	1.58 (0.54–4.62)	0.63 (0.16–2.42)	1.38 (0.50–3.81)	0.52 (0.14–1.87)
MI	2.81 (1.05–7.55)	2.25 (0.65–7.77)	3.55 (1.39–9.10)	3.22 (1.04–9.96)

This table summarizes the relation between mortality and APACHE II score, MI, and each of the three types of advanced life support at any time during the ICU stay (mechanical ventilation: 61.7% of patients; inotropes or vasopressors: 38.3% of patients; hemodialysis: 18.3% of patients). APACHE, Acute Physiology and Chronic Health Evaluation; CI, confidence interval; ICU, intensive care unit; MI, myocardial infarction; OR, odds ratio.
